# Magnetic levitation pumps for cell-free hemoglobin prevention during VV ECMO

**DOI:** 10.1186/s13054-022-03963-9

**Published:** 2022-03-29

**Authors:** Ignazio Condello

**Affiliations:** Perfusion Service, Department of Cardiac Surgery, Anthea Hospital, GVM Care & Research, Via Camillo Rosalba 35/37, 70124 Bari, Italy

Increased plasma concentrations of circulating cell-free hemoglobin (CFH) are supposed to contribute to the multifactorial etiology of acute kidney injury (AKI). In their recent article: “The role of cell-free hemoglobin and haptoglobin in acute kidney injury in critically ill adults with ARDS and therapy with VV ECMO.”, Graw et al. identified a cohort of 1044 ARDS patients with CFH and haptoglobin measurements before initiation of ECMO therapy. They concluded that in critically ill patients with ARDS requiring therapy with VV ECMO, increased plasma concentration of CFH were an independent risk factor for AKI. Among patients with increased CFH concentrations, higher plasma haptoglobin concentrations might protect from CFH-associated AKI [1]. In this context we reported our experience about the effect of Magnetic levitation pump versus Constrained vortex pump on the hemolysis effect during extracorporeal technologies for short time. We reported a pilot study focused on plasma free hemoglobin levels in 40 patients undergoing isolated coronary artery bypass grafting (CABG). The same circuits for minimally invasive extracorporeal circulation (MiECC) were used in all patients. The ECMOLIFE magnetic levitation pump was used in the study group (*n* = 20), and the AP40 Affinity CP centrifugal blood pump was used in the control group (*n* = 20). In the immediate postoperative period, cell-free hemoglobin (CFH) and lactate dehydrogenase (LDH) were significantly lower in the study group than in the control group (10.6 ± 0.7 vs. 19.9 ± 0.3 mg/dL, *p* = 0.034; and 99.2 ± 1.7 vs. 139.2 ± 1.5 IU/L, *p* = 0.027, respectively). Moreover, patients treated with the magnetic levitation pump showed lower creatinine and indirect bilirubin (0.92 vs. 1.29 mg/dL, *p* = 0.030 and 0.6 ± 0.4 vs. 1.5 ± 0.9 mg/dL, *p* = 0.022, respectively) [2]. We think that the materials selection during VV-ECMO with the use of magnetic levitation pump (Fig. 1) for long time could be crucial for the possible reduction of CFH and the indirect bilirubin, however further studies are needed to support our opinion.

**Fig. 1:**
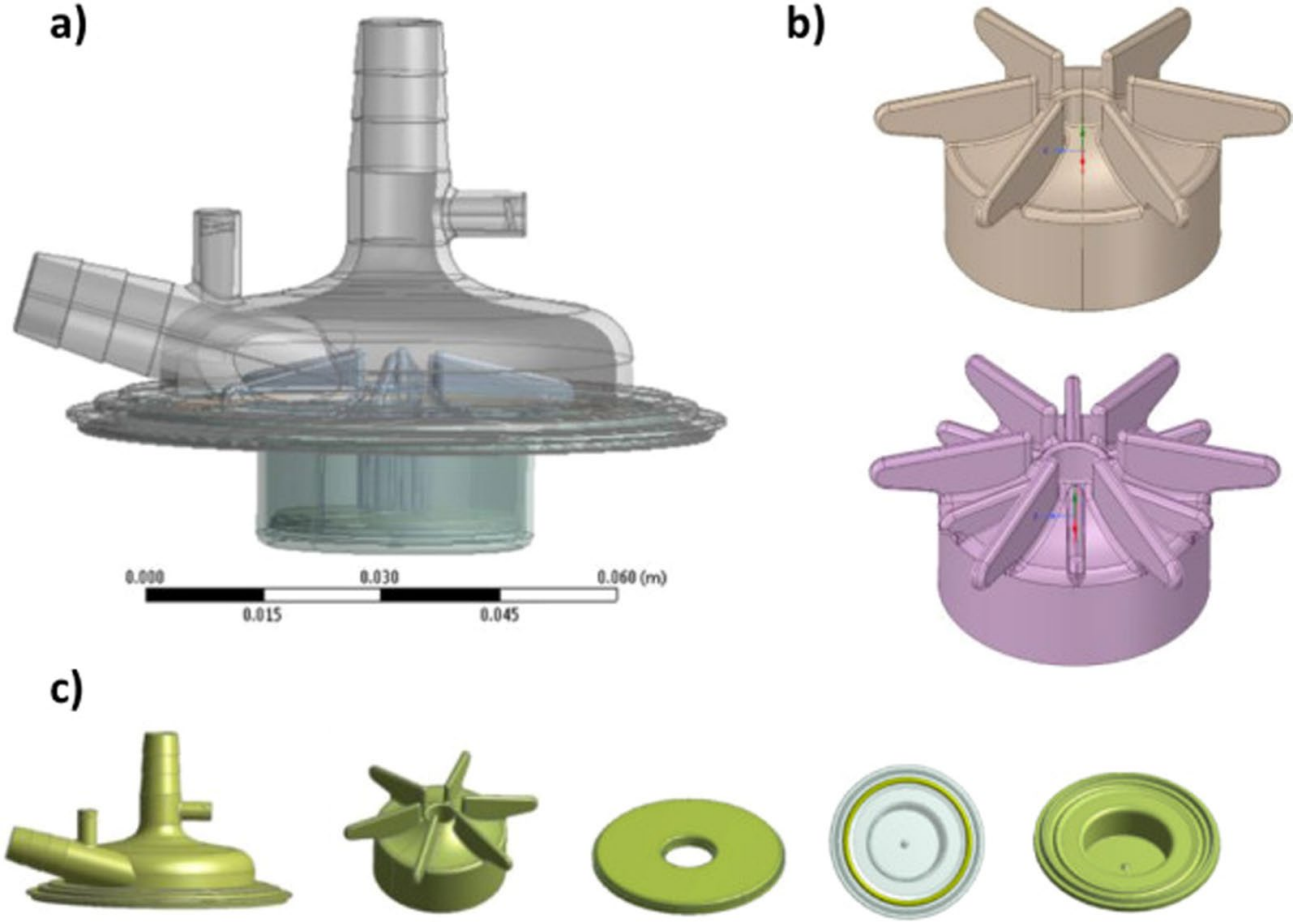
**a** Magnetic levitation pump (ECMOLIFE EUROSETS, MEDOLLA, ITALY). **b** Rotating magnetic disc. **c** ECMOLIFE components

## Data Availability

Not applicable.
